# Draft Genome Sequences of the Bap-Producing Strain Staphylococcus aureus V329 and Its Derived Phage-Resistant Mutant BIM-1

**DOI:** 10.1128/MRA.00500-21

**Published:** 2021-07-15

**Authors:** Lucía Fernández, Ana Catarina Duarte, Beatriz Martínez, Ana Rodríguez, Pilar García

**Affiliations:** a Instituto de Productos Lácteos de Asturias (IPLA-CSIC), Villaviciosa, Asturias, Spain; b DairySafe Group, Instituto de Investigación Sanitaria del Principado de Asturias (ISPA), Oviedo, Spain; Montana State University

## Abstract

This study reports the draft genome sequences of Staphylococcus aureus V329, a Bap-producing strain isolated from a case of subclinical bovine mastitis in Spain, and a derived mutant (BIM-1) resistant to phage phiIPLA-RODI. Comparison of the two genomes revealed that the mutant strain has a point mutation in the gene *tagO*.

## ANNOUNCEMENT

Although Staphylococcus aureus is best known for being an opportunistic pathogen in humans, it can also cause infections in a wide range of animal hosts (1). For instance, this microbe is one of the etiological agents of mastitis in dairy cows, leading to economic losses in this sector and posing a potential risk to human health due to milk contamination. Such infections often exhibit a persistent nature and cannot be successfully cleared with antibiotic treatment.

It is widely accepted that biofilms play a role in chronic, recalcitrant bacterial infections. For most S. aureus strains, the main component of the extracellular matrix is the polysaccharide PIA/PNAG (2). However, some bovine mastitis isolates form strong biofilms rich in the Bap protein (3), a characteristic that has also been observed in other staphylococcal species (4). The first strain identified to produce such biofilms was S. aureus V329, isolated in Spain from a mastitic cow (3). More recently, Duarte et al. (5) isolated a V329-derived mutant, BIM-1, exhibiting resistance to phage phiIPLA-RODI and decreased susceptibility to the lytic protein CHAPSH3b. This study aimed to sequence the genome of V329, kindly provided by A. Toledo-Arana (Instituto de Agrobiotecnología, CSIC-Universidad Pública de Navarra, Spain), and compare it to the mutant BIM-1, recently derived in our laboratory, in order to identify the potential mutation(s) responsible for its phenotype.

Both strains were grown on plates containing TSB (tryptic soy broth; Scharlau Microbiology, Barcelona, Spain) supplemented with 2% agar (Roko, S.A., Llanera, Spain) at 37°C. A single colony was then streaked onto a second plate and incubated overnight under the same conditions. All cells grown on this plate were harvested and stored in Microbank cryovials, which contain beads and a cryopreservative (Pro-Lab Diagnostics UK, UK), until genomic DNA (gDNA) isolation. Then, the beads were washed with extraction buffer containing lysostaphin and RNase A and incubated for 25 min at 37°C. Afterwards, proteinase K and RNase A were added, and the samples were further incubated for 5 min at 65°C. gDNA purification was performed using solid-phase reversible immobilization (SPRI) beads (Beckman Coulter, Brea, CA), and the genomic libraries were prepared using a Nextera XT library prep kit (Illumina, San Diego, CA) according to the manufacturer’s protocol. The resulting libraries were subsequently sequenced on an Illumina HiSeq platform using a 250-bp paired-end protocol. Genome sequencing was provided by MicrobesNG. Default parameters were used for all software utilized for the genome analysis unless otherwise noted. The quality of the reads was assessed using FASTQC v. 0.11.3 (6). Trimming was carried out using Trimmomatic v. 0.39 with a sliding window quality cutoff of Q15 (7). The genomes were assembled *de novo* using SPAdes v. 3.14.1 (8), and the quality of the assembly was assessed using QUAST v. 5.0.2 (9). The genome completeness was assessed using BUSCO v. 5 (10) and the ortholog set “Bacteria” on the gVolante Web server (11). The contigs were reordered using Mauve v. 20150226 (12). Then, sequences shorter than 500 bp were removed prior to genome annotation using the NCBI Prokaryotic Genome Annotation Pipeline v. 5.1 (13). Details regarding the genome assembly and annotation for both strains are shown in Table 1. A comparison of the two genomes using Breseq v. 0.35.5 (14) subsequently revealed that strain BIM-1 had a point mutation in the gene *tagO* (G210E), involved in the biosynthesis of teichoic acids (15). *tagO* deletion is known to result in phage resistance in S. aureus (16). Further work will be necessary to elucidate whether this mutation is responsible for the BIM-1 phenotype.

**TABLE 1:** Genome assembly statistics and annotation features

Feature	Data for strain:	
	V329	BIM-1
No. of reads	610,303	572,792
Assembly statistics		
No. of contigs	50	53
No. of contigs >500 bp	20	19
Largest contig size (bp)	636,777	636,777
Genome size (bp)	2,719,662	2,718,812
G+C content (%)	32.8	32.8
*N*_50_ (bp)	356,410	356,410
No. of Ns per 100 kbp	0	0
Genome completeness (%)	100	100
Annotation features		
No. of genes (coding)	2,539	2,537
No. of tRNAs	53	53
No. of rRNAs	12	11

### Data availability.

The whole-genome shotgun projects corresponding to S. aureus V329 and BIM-1 have been deposited at DDBJ/ENA/GenBank under accession numbers JAGTJH000000000 and JAGTJI000000000, respectively. The version described in this article is the first version. The corresponding files containing the raw reads have been deposited at NCBI under BioProject accession number PRJNA723827.
